# New Insights into Chemical Profiles and Health-Promoting Effects of Edible Mushroom *Dictyophora indusiate* (Vent ex. Pers.) Fischer: A Review

**DOI:** 10.3390/jof11010075

**Published:** 2025-01-18

**Authors:** Yogesh Kumar, Baojun Xu

**Affiliations:** 1 Department of Biotechnology, Mehsana Urban Institute of Sciences, Ganpat University, Mehsana 384012, Gujrat, India; yogeshkumarbiotech@gmail.com; 2 Food Science and Technology Program, Department of Life Sciences, BNU-HKBU United International College, Zhuhai 519087, China

**Keywords:** *Dictyophora indusiate*, *Phallus indusiate*, mushrooms, monoterpene, alkaloids, sesquiterpenes

## Abstract

Mushrooms are valued for their culinary and medicinal benefits, containing bioactive compounds like polysaccharides, terpenoids, phenolics, lectins, and ergosterols. This review aims to encourage research on *D. indusiata* by summarizing its chemistry, health benefits, pharmacology, and potential therapeutic applications. Molecules from *D. indusiata* offer anti-diabetic, antioxidant, anti-tumor, hepatoprotective, and anti-bacterial effects. In particular, polysaccharides from *Dictyophora indusiata* (DIP) enhance immune function, reduce oxidative stress, and promote gut health as prebiotics. DIP shows neuroprotective effects by reducing oxidative damage, improving mitochondrial function, and regulating apoptosis, making them beneficial for neurodegenerative diseases. They also activate immune responses through TLR4 and NF-κB pathways. Additionally, compounds like dictyophorines and quinazoline from *D. indusiata* support nerve growth and protection. Mushrooms help regulate metabolism and improve lipid profiles, with potential applications in managing metabolic disorders, cancer, cardiovascular diseases, diabetes, and neurodegenerative conditions. Their wide range of bioactive compounds makes *D. indusiata* mushrooms functional foods with significant therapeutic potential.

## 1. Introduction

Medicinal fungi are valuable sources of bioactive compounds and widely used as functional foods. *Dictyophora indusiata* (Vent.) Fisch., also known as *Phallus indusiatus*, is an edible and medicinal mushroom belonging to the Phallaceae family in the Agaricomycetes class and Basidiomycetes division of fungi. Although the name *Phallus indusiatus* is accepted in recent taxonomy, most scientific literature refers to it as *D. indusiata*. This saprophytic fungus thrives on decayed wood and rich soil in tropical regions such as Africa, Asia, Australia, and the Americas. It is highly valued for its food and medicinal properties in countries like China, where it grows in bamboo forests. *D. indusiata* is renowned for its elegant morphology, earning it names like the veiled lady or queen of mushrooms. Other common names include bamboo mushroom and bridal veil fungus. The common name aligns with its distinctive fruiting body, which features a conical cap, a stalk, and a net-like indusium resembling a skirt [1]. *D. indusiata* is rich in nutritional value, containing up to 47% carbohydrates, 29% crude fiber, and 6% protein by dry weight [2]. Additionally, it provides essential amino acids, vitamins, and minerals. Beyond its nutritional content, the focus has shifted to its pharmacologically active components, particularly its polysaccharides, which have therapeutic potential. Its medicinal potential is being explored, with key bioactive components including polysaccharides, particularly β-(1→3)-D-glucan, and small molecules like terpenoids and alkaloids. These compounds exhibit antioxidant, anti-inflammatory, and signaling properties, with promising applications in cancer, immunotherapy, neurodegenerative, and chronic inflammatory diseases [1].

The water extract of *D. indusiata* exhibited antioxidant activity in vitro through DPPH, hydroxyl, and ABTS radical scavenging assays. In *Caenorhabditis elegans*, the extract enhanced antioxidant capacity, prolonged lifespan under stress, and reduced lipid deposition and triglyceride levels. It regulated key genes involved in lipid metabolism, indicating its potential for antioxidant and lipid-lowering effects [3]. Currently, *D. indusiata* is valued for two main purposes: its stipe is used as a delicacy, and it possesses various medicinal properties, including anti-tumor, cardiovascular-protective, antioxidant, immunomodulatory, and anti-bacterial effects, along with benefits for eye health and mental well-being [4,5,6]. Although *D. indusiata* is now cultivated in southern China, challenges persist in its cultivation, resource utilization, and development. The fruiting bodies mature rapidly within hours, requiring immediate harvest to prevent autolysis and waste. Typically, only the stipe is consumed, while the cap, indusia, and volva are discarded, resulting in significant resource wastage [7].

**Figure 1:**
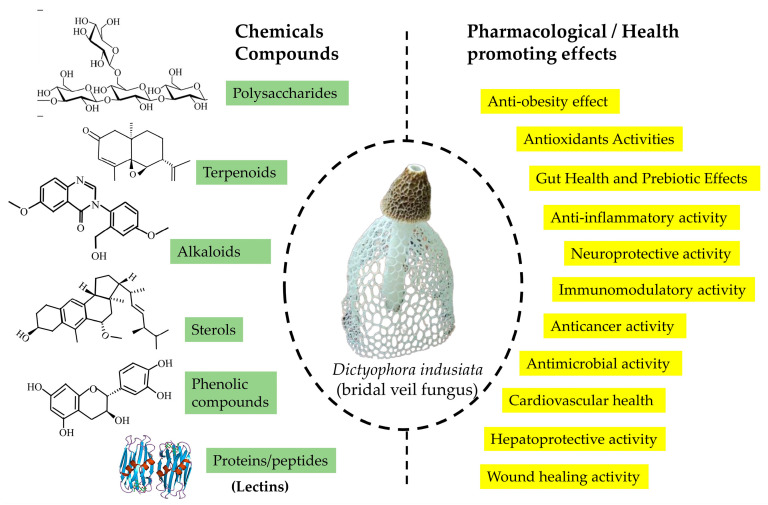
Overview on chemical profiles and health-promoting effects of *D. indusiata*.

Although the polysaccharides of *D. indusiata* are well-studied, other bioactive compounds like terpenoids and alkaloids have received comparatively less attention. The abundance of polysaccharides in the mature fruiting body has made them the primary focus of research. *D. indusiata* polysaccharides (DIP) have shown notable anti-tumor, antioxidant, anti-inflammatory, immune-enhancing, and anti-diabetic effects. However, the smaller bioactive compounds in *D. indusiata* remain largely underexplored compared to its polysaccharides [1,2,8]. This review provides new insights into the mushroom’s chemical profile, emphasizing these underexplored compounds and their potential health benefits, including antioxidant, anti-inflammatory, immunomodulatory, wound healing, hepatoprotective, cardiovascular, and therapeutic properties. It particularly focuses on their roles in anti-bacterial activity, cancer treatment, neurodegenerative disorders, anti-diabetic effects, hepatoprotection, and lipid metabolism, while also addressing potential toxic effects (Figure 1).

## 2. Overview of Chemical Profiles of *Dictyophora indusiata*

*D. indusiata*, a medicinal mushroom, contains various bioactive compounds, including polysaccharides, sterols, phenolic compounds, alkaloids, and terpenoids. These chemical classes are known for their therapeutic properties, such as antioxidant, anti-inflammatory, and antimicrobial effects (Table 1).

### 2.1. Polysaccharides

**Figure 2:**
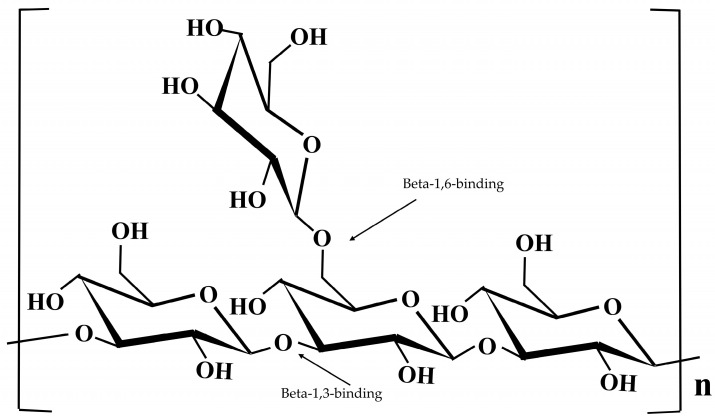
Monomer structure of polysaccharides as 1,3-β-glucans with 1,6-β-glucans branch. The arrow indicates the beta-1,3-glycosidic bond in a chain and the beta-1,6-glycosidic bond at the branch point.

Polysaccharides from *D. indusiata* fruiting bodies were extracted and purified, with a molecular mass of 536 kDa and a total sugar content of 97.6%. Monosaccharide composition analysis showed that DIP consists primarily of D-glucose (90.7%). Structural analysis using ^13^C-NMR revealed that DIP is a homogeneous β-(1→3)-D-glucan with β-(1→6)-glucosyl side branches, as shown in Figure 2. The beta-configuration was confirmed by key peaks at 103.5 ppm (C1), while signals at 86.6 ppm (C3) and 61.7 ppm (C6) indicated 1,3-beta-linkages. Branching was observed at C2′, C3′, and C5′, further confirmed by C6 peaks, detailing DIP’s complex structure [20].

In a study, it was found that the fruiting bodies of *D. indusiata* are rich in heycarbohydrates (46.89%) and crude fibers (28.65%), with a lower crude protein content of 6.07%. Soluble polysaccharides constitute 37.5% of the fruiting bodies, representing approximately 80% of the total polysaccharides. This suggests a significant polysaccharide-based medicinal effect, while peptidoglycan-containing fractions (D1, D2, D4, and D6) may also contribute to therapeutic properties. In contrast, fractions D3 and D5 exhibit glycoprotein characteristics. Monosaccharide analysis revealed fraction D1 as a glucogalactan, D3 as a riboglucan, and D5 and D6 as mannogalactans. Fraction D4, with 92.5% myo-inositol, is nearly pure myo-inositol, indicating potential specialized uses [2].

In a study, acid- and alkali-soluble polysaccharides (DIP I and II) were extracted from the fruiting bodies of *D. indusiata*. DIP I was composed of glucose (Glc), fructose (Fru), and mannose (Man), while DIP II contained only Glc and Fru. Glc was the dominant monosaccharide in both DIPs, accounting for more than 60%, forming the backbone of the polysaccharide chain. Glycosidic linkage analysis revealed Glc 1→6 linkages in DIP II. Hydrolysis by trifluoracetic acid and subsequent monosaccharide identification showed that DIP I had molar percentages of Glc and Fru at 66.2% and 14.5%, respectively, and DIP II at 86.4% and 13.6%. Mannose was present only in DIP I. The structural analysis indicated that Glc forms the backbone for both DIPs, with differences in linkage ratios between them [21].

PD3, a water-soluble (1→3)-β-D-glucan with (1→6)-β-glucopyranoside side branches, was isolated from *D. indusiata*. Its molecular structure and conformation were analyzed using GC, FTIR, NMR, and microscopy techniques. PD3 forms a triple helical chain in water and undergoes helix–coil transition at higher NaOH concentrations compared to similar polysaccharides, self-assembling into fibrous aggregates [22].

### 2.2. Terpenoids

**Figure 3:**
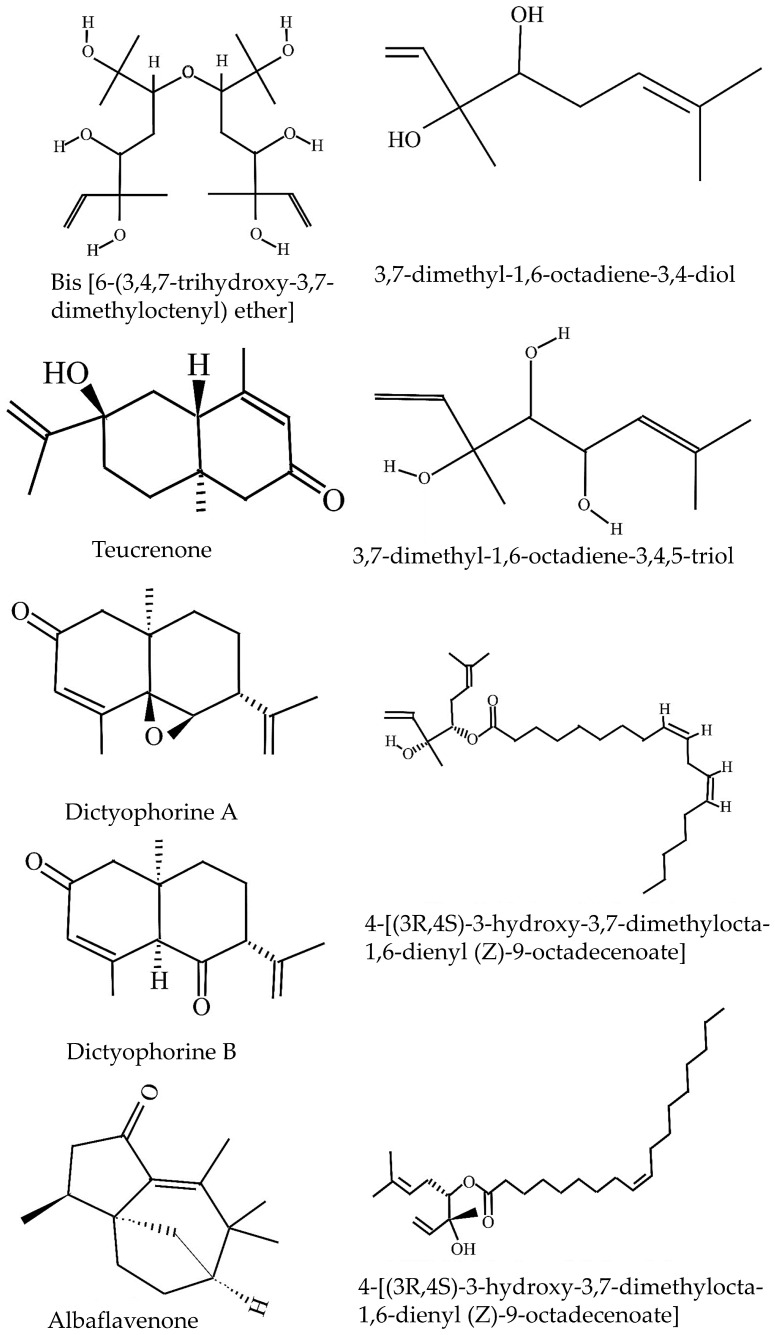
Structure of different terpenoids from *D. indusiata*.

The isolation and characterization of monoterpenoids *D. indusiata* were documented [23]. The air-dried fruiting bodies of *D. indusiata* (1.2 kg) were extracted with 85% ethanol, and the solvent was concentrated under reduced pressure. Partitioning between ethyl acetate and water resulted in a residue (20.2 g), which was further fractionated using silica gel chromatography and HPLC. This yielded four compounds. Among the isolated compounds were five monoterpene alcohols, with the first identified as 3,7-dimethyl-1,6-octadiene-3,4-diol, confirmed to have a (3R,4S) absolute configuration. The remaining four were identified as 4-[(3R,4S)-3-hydroxy-3,7-dimethylocta-1,6-dienyl (Z)-9-octadecenoate], 4-[(3R,4S)-3-hydroxy-3,7-dimethylocta-1,6-dienyl(9Z,12Z)-9,12-octadecadienoate], 3,7-dimethyl-1,6-octadiene-3,4,5-triol, and bis[6-(3,4,7-trihydroxy-3,7-dimethyloctenyl) ether]. These structures were determined through comprehensive spectral and chemical analysis. Structure of different terpenoids from *D. indusiata* are given in Figure 3.

Air-dried fruiting bodies of *D. indusiata* (1.2 kg) were extracted using 85% ethanol, and the solvent was concentrated under reduced pressure [15]. The extract was partitioned between ethyl acetate and water, yielding a residue (20.2 g). This residue was fractionated through repeated silica gel column chromatography (CC) and HPLC, resulting in the isolation of two compounds: 1 (246 mg, crystalline, mp 39–40 °C) and 2 (8 mg, amorphous). Two novel eudesmane-type sesquiterpenes, dictyophorines A and B, along with the known compound teucrenone, were identified.

Thirty-five grams of dried *D. indusiata* fruiting bodies was subjected to Soxhlet extraction with dichloromethane as the solvent for 24 h [17]. After extraction, the concentrate underwent silica gel column chromatography, yielding 1.7 mg of a crude product. Through FTIR, GC-MS, HR-FIMS, and NMR analyses, the isolated compound was identified as the sesquiterpene antibiotic albaflavenone. The content of albaflavenone in the dried fruiting bodies was determined to be approximately 0.0063%. The compound exhibited an earthy, camphor-like odor, showcasing its potential as a bioactive molecule with antimicrobial properties. Similarly, researchers isolated two novel sesquiterpenoids, designated as 9,10-dihydroxy-albaflavenone (1) and 5-hydroxy-albaflavenone (2), from *D. indusiata* [24]. The structures and absolute configurations of these compounds were elucidated through NMR, ECD, and HR-ESI-MS analysis.

### 2.3. Alkaloids

**Figure 4:**
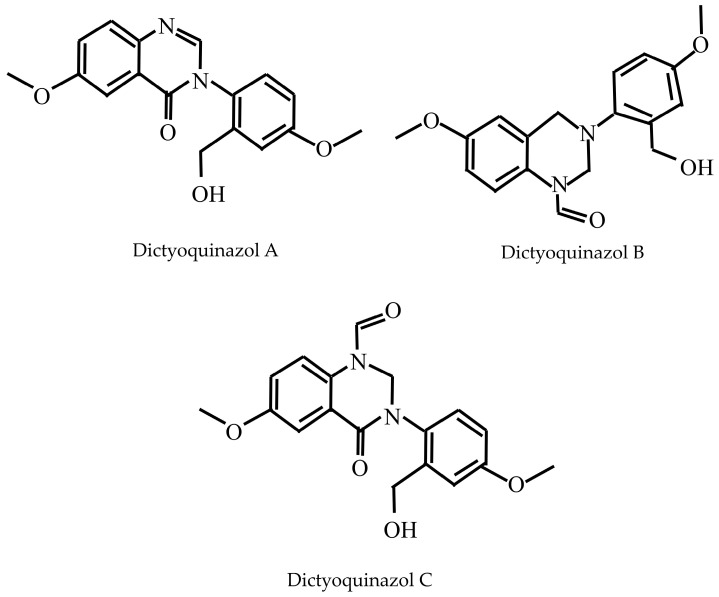
Structure of alkaloids isolated from *D. indusiata*.

A fresh *D. indusiata* (1.2 kg) was collected and methanolic extraction of the mushroom was performed followed by column chromatography, and HPLC yielded three neuroprotective quinazoline compounds (Figure 4): dictyoquinazols A, B, and C. Dictyoquinazols B and C existed as inseparable rotamer mixtures, confirmed by NMR and NOESY spectra analysis [16].

### 2.4. Phenolic Compounds

**Figure 5:**
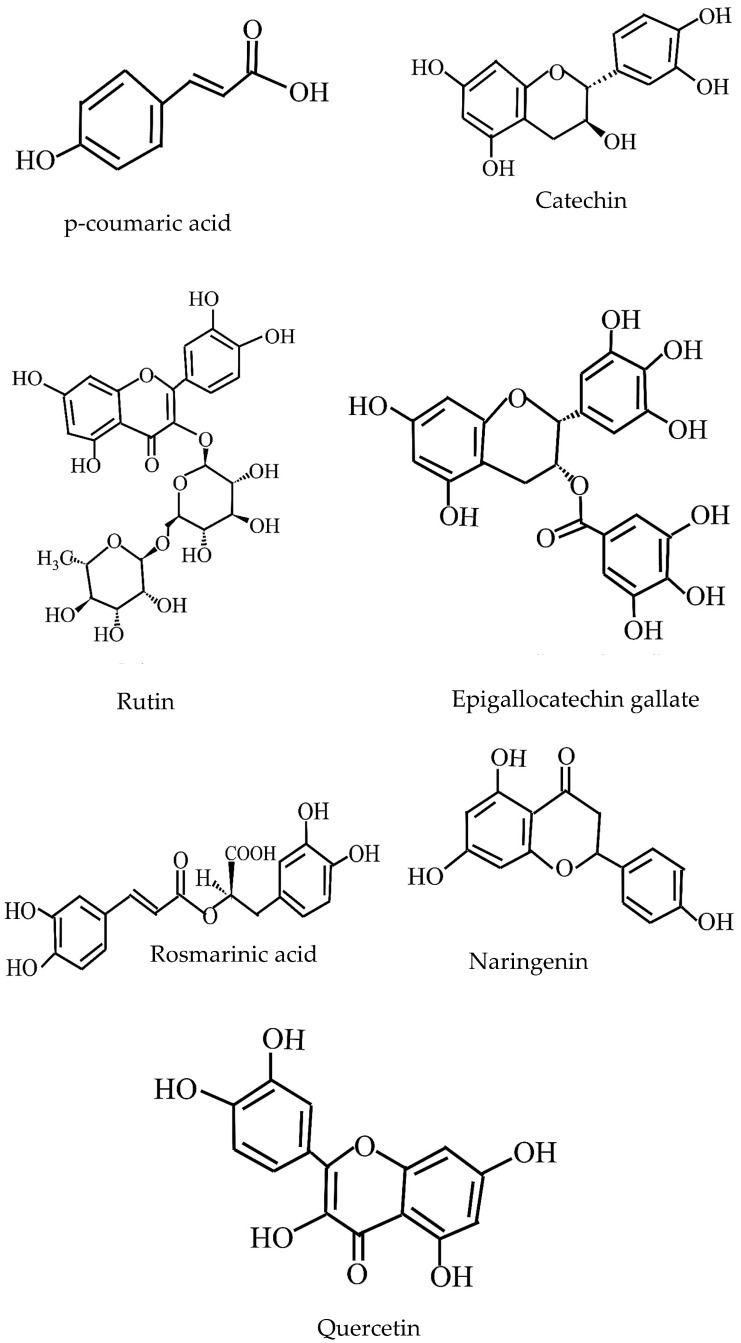
Structure of phenols isolated from *D. indusiata*.

The crude water extracts from the immature stage of *D. indusiata* yielded 21.11%, 51.48%, and 10.15% of peel and green mixture (PGW), core (CW), and whole (WW) extracts, respectively [10]. The primary bioactive components were phenolic compounds (Figure 5). Catechin (68.761 mg/g) was the dominant polyphenol in CW, while p-coumaric acid was the major polyphenol in PGW and WW. Other identified polyphenols included rutin, rosmarinic acid, quercetin, naringenin, and epigallocatechin gallate (EGCG).

### 2.5. 5-(Hydroxymethyl)-2-furfural

**Figure 6:**
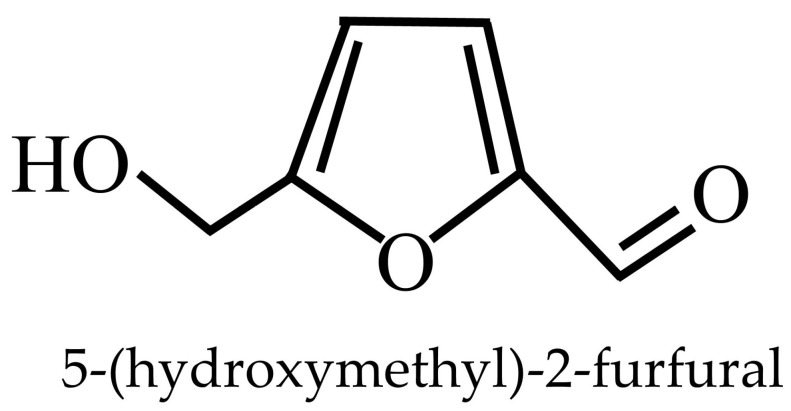
Structure of 5-(hydroxymethyl)-2-furfural.

The evaluation of tyrosinase inhibition by methanolic extracts of *D. indusiata* identified the bioactive compound as 5-(hydroxymethyl)-2-furaldehyde (HMF) (Figure 6). Kinetic studies revealed HMF to be a non-competitive inhibitor for L-DOPA oxidation. Freeze-dried mushroom extracts were partitioned with hexane, CH_2_Cl_2_, EtOAc, and BuOH, with the active EtOAc fraction (3 g) yielding HMF after chromatographic purification. The structure of HMF was confirmed through ^1^H NMR, ^13^C NMR, and mass spectrometry, showing that it matched the authentic standard [25].

### 2.6. Sterols

**Figure 7:**
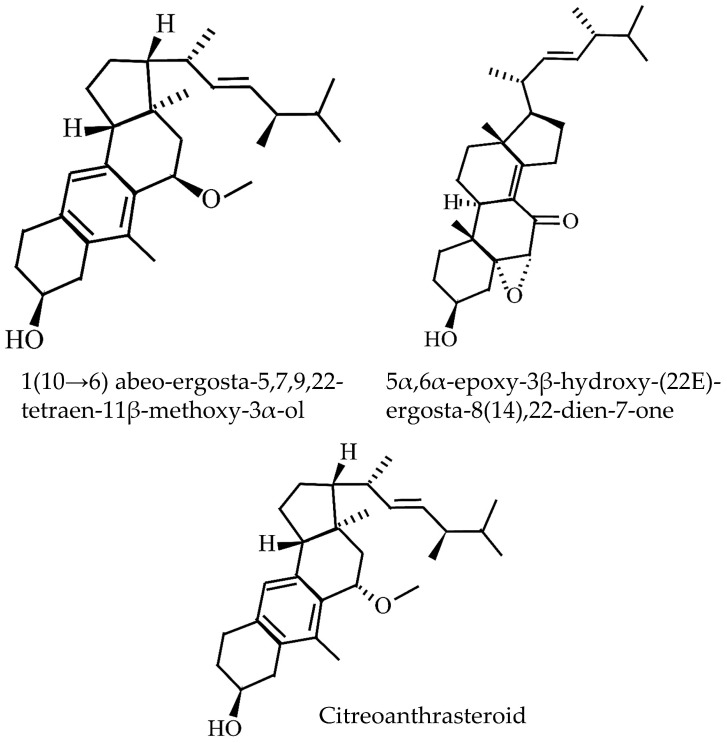
Structure of sterols identified from *D. indusiata*.

Three ergostane-type steroids (Figure 7), namely compounds 15, 16, and 17, from *D. indusiata* demonstrated significant anti-inflammatory properties [26]. Compounds 15 [1(10→6) abeo-ergosta-5,7,9,22-tetraen-11β-methoxy-3α-ol] and 16 (citreoanthrasteroid) were ergosterol derivatives with rearranged tetracyclic skeletons, differing in their stereochemistry; both had a methoxy group at C-11, with compound 15 having it on the same face as the hydroxyl group and compound 16 on the opposite face. Compound 17 [5α,6α -epoxy-3β-hydroxy-(22E)-ergosta-8(14),22-dien-7-one], identified as an epoxy-7-sitosterol, was noted for its epoxy and carbonyl groups, which appeared to be crucial for its biological activity.

### 2.7. Proteins/Peptides

A lectin from *D. indusiata* was isolated through saline extraction, ammonium sulfate precipitation, and chromatography. SDS-PAGE revealed it as a single 53 kDa subunit. The lectin agglutinated rabbit and human erythrocytes, as well as mouse lymphocytes and tumor cells. Hemagglutination was inhibited by lactose and L-fucose [27].

## 3. Health-Promoting Effects of *Dictyophora indusiata*

*D. indusiata* exhibits health-promoting effects by reducing inflammation, improving gut microbiota balance, protecting liver function, exerting anticancer, antimicrobial, immunomodulatory, and neuroprotective effects, and mitigating oxidative stress, making it a potential candidate for therapeutic use.

### 3.1. Antioxidants Activities

The water extract of *D. indusiata* demonstrates significant in vitro antioxidant activity, as shown by DPPH, hydroxyl radical, and ABTS cation radical scavenging assays, with IC50 values of 2.36, 1.44, and 0.86 mg/mL, respectively [3,28]. Using *Caenorhabditis elegans* as a model, the extract at various doses (1.0, 0.5, and 0.25 mg/mL) enhanced oxidative and heat stress tolerance, extending lifespan by 54.6% and 19.8%. Additionally, lipid deposition and triglyceride levels decreased significantly, potentially through the modulation of fat metabolism genes such as daf-2, sbp-1, and nhr-49. These findings suggest the extract’s potential in antioxidant activity and lipid metabolism improvement [3].

A purified water-soluble polysaccharide (PPS) from *D. indusiata*, primarily composed of glucose (98.58%), was characterized using gel chromatography (Sephadex G-200), IR spectroscopy, and GC–MS. PPS exhibited antioxidant activity in vitro across multiple assays, including reducing power, hydroxyl, superoxide radical, and DPPH scavenging tests. These findings suggest that PPS is a promising natural antioxidant with potential therapeutic applications in mammalian systems, offering a novel approach to antioxidant therapy [29]. Phenolic compounds are also reported from *D. indusiata* that might also contribute to alleviating metabolic ROS. Phenolic and polyphenolic compounds, alone or combined with vitamins like carotenoids, vitamin E, and vitamin C, act as antioxidants, protecting human tissues from oxidative stress [30].

### 3.2. Gut Health and Prebiotic Effects

Despite the well-documented biological activities of *D. indusiata* polysaccharides (DIP), their influence on gut microbiota restoration has remained largely unexplored. DIP modulates the gut microbiota composition and restores intestinal barrier function following broad-spectrum antibiotic-induced dysbiosis. Using the Illumina MiSeq platform, the study revealed that DIP administration reversed the antibiotic-driven microbial imbalance, increasing beneficial bacteria such as Lactobacillaceae and Ruminococcaceae. Additionally, DIPs reduced endotoxemia and pro-inflammatory cytokines while enhancing the expression of tight-junction proteins, crucial for intestinal barrier integrity. These results underscore the therapeutic potential of DIPs in restoring gut health, improving barrier function, and mitigating inflammation [31].

Researchers successfully isolated crude polysaccharide (DIP) from the fruiting body of *D. indusiata*, with a total sugar content of 96.66%. Monosaccharide composition analysis revealed that the bioactive components were primarily glucose (59.84%), mannose (23.55%), and galactose (12.95%). Dysbiosis was induced in BALB/c mice using broad-spectrum antibiotics, clindamycin and metronidazole, to investigate the protective effects of DIP. Gut bacterial diversity disruption due to antibiotics and its recovery following DIP administration (0.2 mg/0.2 mL) were assessed through PCR-DGGE and further analyzed using the 16S rRNA Illumina MiSeq technique, providing a comprehensive understanding of DIP’s role in restoring gut microbial balance [31].

This work was supported by further research on high-fat-diet-induced obesity in mice [11]. HFD is known to disrupt the microbial balance, while dietary fibers and polysaccharides have been shown to positively influence gut microbial communities [32]. DIP supplementation significantly altered the gut microbiota composition, enhancing bacterial diversity and richness in a dose-dependent manner compared to the HFD-alone group. Notably, the Firmicutes-to-Bacteroidetes ratio was restored and bacterial perturbations at the phylum and class levels were reversed with DIP treatment.

The high-fat diet causes a detrimental effect on intestinal integrity and systemic inflammation, driven by elevated pro-inflammatory cytokines and endotoxemia (LPS in blood bloodstream) through TLR4 signaling activation [33]. As expected, HFD-induced obese mice demonstrated increased endotoxin levels and reduced tight junction protein (TJP) expression, leading to intestinal barrier dysfunction. However, DIP supplementation effectively reduced LPS levels and upregulated TJP expression, indicating its potential to preserve intestinal health and mitigate inflammation [11].

### 3.3. Anti-Inflammatory Activity

Previous studies have shown that intestinal injury and inflammation lead to increased expression of pro-inflammatory cytokines [34], which negatively impact intestinal mucosal integrity and function [35]. Antibiotic-induced dysbiosis in mouse models exacerbates intestinal inflammation, resulting in elevated levels of pro-inflammatory cytokines such as TNF-α, IL-1β, MCP-1, and IFN-γ [36,37]. In line with these findings, their study observed an increased abundance of Bacteroidetes and Proteobacteria in antibiotic-treated groups, which are linked to heightened pro-inflammatory cytokine production. However, administration of *D. indusiata* polysaccharides (DIP) significantly reduced these cytokine levels and alleviated the inflammatory response by modulating gut microbiota. These results suggest that DIPs effectively mitigate antibiotic-induced inflammation and enhance immune function [31].

Another study highlights the potential of DIP, a natural polysaccharide, as an anti-inflammatory agent targeting the TLR4/NF-κB signaling pathway and NLRP3 inflammasome activation. Chronic inflammatory and autoimmune diseases, such as IBD and rheumatoid arthritis, involve dysregulated inflammasome activity [38,39]. DIP was shown to repress TLR4 expression and inhibit NF-κB activation by preventing IκB-α phosphorylation and NF-κB-p65 nuclear translocation in LPS-primed macrophages. It also reduced the production of pro-inflammatory cytokines like IL-1β, IL-18, and IL-6, thereby attenuating the inflammatory response [40]. Additionally, DIP inhibited the assembly of the NLRP3 inflammasome, decreasing NLRP3 and pro-caspase-1 expression and preventing mitochondrial ROS production, a key factor in NLRP3 activation [41]. DIP’s non-dose-dependent effect, with higher concentrations promoting inflammation, warrants further investigation [19]. Its structural similarity to lentinan, which has demonstrated therapeutic effects in an IBD model, suggests DIP may have similar benefits [42]. However, further research is needed to evaluate its efficacy within the gastrointestinal environment. Overall, DIP presents a promising candidate for treating chronic inflammatory diseases by modulating key inflammatory pathways and suppressing inflammasome activation [43]. In another report, albaflavenone, a tricyclic sesquiterpene, demonstrated anti-inflammatory properties by inhibiting TNF-α and NO secretion to different extents [24].

### 3.4. Neuroprotective Activity

*D. indusiata*, a traditional medicinal mushroom in China, has shown potential for treating inflammatory and neural conditions. This study highlights the antioxidant and neuroprotective properties of *D. indusiata* polysaccharides (DIP) using *Caenorhabditis elegans* models. DiPS increased survival rates, reduced oxidative stress markers (ROS, MDA), enhanced SOD activity, and restored mitochondrial function. Furthermore, DIP involved the DAF-16/FOXO transcription factor in stress response and alleviated chemosensory dysfunction in neurodegenerative disease models. These findings offer significant evidence of DIP therapeutic potential for neurodegenerative diseases, providing a pharmacological basis for its use in mitigating oxidative stress and neuroprotection [44]. The neuroprotective properties of *D. indusiata* can be attributed to the presence of dictyophorines and polysaccharides. Notably, dictyophorines A and B were found to promote nerve growth factor (NGF) synthesis in astroglial cells, indicating their potential neuroprotective and therapeutic properties [15]. The quinazoline compounds also exhibited neuroprotective effects against glutamate- and NMDA-induced excitotoxicity in primary mouse cortical neurons [16]. The neuroprotective effects of polysaccharides have been also demonstrated in Sprague Dawley (SD) rats. In arsenic-exposed rats, *D. indusiata* polysaccharide (DIP) has been shown to enhance spatial learning and memory, which were otherwise impaired due to arsenic exposure [45]. DIP exhibits neuroprotective effects by mitigating arsenic-induced damage, possibly by reversing the dysregulation of synaptic proteins and restoring glutamatergic neurotransmission, improving learning and memory in arsenic-exposed rats. DIP also attenuates apoptosis by regulating apoptosis-related proteins and mitigating mitochondrial dysfunction. Furthermore, DIP restores normal energy metabolism pathways by enhancing ATP production and reducing oxidative stress. By modulating key signaling pathways such as Ras, MAP kinase, and Wnt, DIP protects against neuronal apoptosis and synaptic dysfunction [45].

### 3.5. Immunomodulatory Activity

DIP significantly stimulates the proliferation of RAW 264.7 cells in a dose- and time-dependent manner, particularly at concentrations of 25–200 μg/mL [19]. DIP enhances the production of nitric oxide (NO) and pro-inflammatory cytokines IL-1β, IL-6, and TNF-α. This effect is associated with the up-regulation of mRNA expression for iNOS, IL-1β, IL-6, and TNF-α, confirming that DIP promotes macrophage activation through gene regulation. Additionally, DIP’s effects were found to be mediated by TLR4, a key receptor involved in macrophage activation, as indicated by the inhibition of NO and cytokine production when anti-TLR4 mAb was applied. Specific binding of DIP to macrophages was demonstrated, and TLR4 was confirmed as the major receptor facilitating this process. Furthermore, DIP activated the NF-κB signaling pathway, upregulating NF-κB p65, further emphasizing its role in promoting immune responses. In a similar report, DIP enhances immunomodulatory activity in RAW 264.7 macrophages, promoting cell multiplication and increasing nitric oxide and cytokine production, including TNF-α, IL-1, IL-6, and IL-12 [18]. It shows a dose-dependent immune response with no toxic effects, suggesting potential biomedical applications. The water-soluble polysaccharide (DI) extracted from *D. indusiata* was a β-(1→3)-glucan with β-(1→6)-glucosyl side branches and a triple-helical structure.

### 3.6. Anticancer Activity

The polysaccharide PD3, isolated from *D. indusiata*, was identified as a (1→3)-β-D-glucan with (1→6)-β-glucopyranoside side chains. The regenerated form, RPD3, retained its triple-helical structure, as evidenced by Congo red testing, but had lower chain tightness compared to PD3. This structural modification, induced by a denaturation-renaturation process, made RPD3 more susceptible to disruption in NaOH solution. As a result, more active sites were exposed to RPD3, potentially increasing its bioactivity by enhancing its ability to interact with immune cell receptors. In vitro, assays revealed that neither PD3 nor RPD3 demonstrated direct cytotoxicity against S-180 tumor cells, but in vivo experiments showed significant anti-tumor effects. Notably, RPD3 exhibited greater tumor inhibition compared to PD3, showing a dose-dependent increase in efficacy (*p* < 0.05) [14]. Both polysaccharides improved immune organ indices, upregulated key cytokines (IL-2, IL-6, and TNF-α), and stimulated immune responses, confirming their role as potent immunopotentiators rather than direct cytotoxic agents [14,46]. This enhancement in bioactivity could be attributed to the denaturation process, as previous studies have indicated that lower concentrations of NaOH during denaturation preserve bioactivity by exposing more functional groups. The observed immune stimulation suggests that the anti-tumor effects of these polysaccharides are primarily mediated through immune system activation rather than direct tumor cell destruction [47]. These findings highlight the potential of RPD3 as a promising candidate for enhancing immune function and combating tumors through immunomodulation.

Apart from this, *D. indusiata* has demonstrated promising anti-cancer properties, specifically inducing cytotoxicity in cholangiocarcinoma (CCA) cell lines. In this study, extracts from three types of Dictyophora species—Chinese bamboo mushrooms (Ch-DTP), Short skirt bamboo mushrooms (Th-DTP), and Orange skirt bamboo mushrooms (Or-DTP)—were tested against CCA cells. Ch-DTP showed the strongest cytotoxic effect, significantly reducing cell viability in all CCA cell lines, even at low concentrations. In contrast, Th-DTP and Or-DTP displayed moderate to limited effects at lower doses but exhibited stronger cytotoxicity at higher concentrations. The results indicate that Dictyophora extracts may induce apoptosis, as treated CCA cells showed signs of programmed cell death, such as cytoplasmic shrinking and nuclear condensation. Importantly, normal fibroblast cells were not affected, suggesting minimal adverse effects [48].

### 3.7. Antimicrobial Activity

Albaflavenone is a sesquiterpenoid compound originally identified in Streptomyces species (e.g., *Streptomyces albidoflavus*, *S. coelicolor*). It is biosynthesized from epi-isozizaene and has been studied for its antibiotic and antimicrobial properties. Albaflavenone’s unique structure, featuring an oxidized sesquiterpene backbone, contributes to its biological activity, making it a subject of interest in natural product research [49]. Albaflavenone has been also isolated from *D. indusiata* mushrooms [17,24]. Dried fruiting bodies of *D. indusiata* were used in the study to evaluate the antimicrobial activity of its hot water extract (WE) through the agar well diffusion method. At 200 mg/mL, WE displayed broad-spectrum antimicrobial effects against both bacteria and fungi, with Alcaligenes faecalis being the most susceptible bacterium and Escherichia coli the least. Among fungi, Candida albicans was the most sensitive to the extract [12].

### 3.8. Anti-Obesity Effect

Obesity, marked by systemic inflammation, is linked to the onset of various chronic diseases, including diabetes, cardiovascular issues, liver inflammation, metabolic disorders, and cancer [50]. Excessive lipid accumulation plays a critical role in obesity development. A high-fat diet (HFD) significantly increases body weight, fat deposition, and liver weight [11]. However, treatment with *D. indusiata* polysaccharide (DIP) significantly reduced body weight gain, fat accumulation, and liver weight, suggesting its protective effects against HFD-induced obesity. Elevated glucose and insulin levels, commonly seen in HFD-induced obesity [51], were also mitigated by DIP supplementation. These results align with prior studies that associate obesity with enlarged adipocytes and the upregulation of adipogenic and lipogenic genes [51,52].

High-fat emulsion-induced obesity led to disturbances of lipid profile, increased hepatic enzyme activities, and oxidative stress markers. Supplementation with WPS (water-soluble polysaccharides) from *D. indusiata* effectively restored normal lipid levels, liver enzyme activities (ALT, AST, ALP, CK, and LDH), and antioxidant status (GSH-Px, SOD, CAT, and T-AOC), demonstrating its potential in mitigating obesity-related metabolic and oxidative stress disturbances [13]. DIP effectively reduced adipocyte size and the expression of these genes in a dose-dependent manner. Furthermore, DIP lowered pro-inflammatory cytokines (TNF-α, IL-1β, IL-6, and MCP-1) and elevated anti-inflammatory cytokines (IL-4, IL-10), counteracting obesity-induced inflammation [53]. DIP also improves liver function by decreasing liver injury markers (ALT, AST), triglycerides, and free fatty acids. These findings suggest that DIP polysaccharide holds promise as a therapeutic agent for managing obesity-related inflammation and liver dysfunction [11].

### 3.9. Cardiovascular Health

A key process driving hyperlipidemia is atherosclerosis, which involves both inflammatory and immune responses. In its initial stages, oxidative stress—caused by factors like elevated lipid levels, smoking, diabetes, or hypertension—leads to endothelial dysfunction. This is followed by the oxidation of low-density lipoprotein (LDL) within the blood vessels. The oxidized LDL (ox-LDL) accumulates and engages scavenger receptors on macrophages, prompting their activation and subsequent uptake of ox-LDL. This progression is central to the development of atherosclerosis [54]. Primary care physicians dedicate significant time and effort to preventive medicine, with a focus on managing conditions like hyperlipidemia to reduce the risk of cardiovascular disease (CVD). Hyperlipidemia is the second most common chronic condition treated, following hypertension. It is well known that hyperlipidemia is a major risk factor for CVD. This condition involves elevated levels of cholesterol, triglycerides, or both, and while it can result from genetic factors, it is more often an acquired condition [55]. The presence of β-sitosterol in *D. indusiata* has been also observed [24]. The cardiovascular protective effects of β-sitosterol, a key component found in cholesterol-lowering functional foods, have been attributed to its antioxidant properties and its ability to reduce cholesterol levels [56]. It was also observed that TNF-α treatment led to increased NF-κB phosphorylation in HAECs, indicating NF-κB activation. However, treatment with β-sitosterol (0.1–200 µM) significantly reduced NF-κB phosphorylation, suggesting that its anti-inflammatory effects in vitro are partially mediated through NF-κB inactivation. This aligns with findings by Moreno, who also demonstrated that β-sitosterol reduced NF-κB activation in PMA-stimulated macrophages [57,58]. Numerous animal studies have shown that the intake of plant sterols inhibits the progression of atherosclerosis. It is well established that after consuming plant sterol-enriched foods, plasma levels of plant sterols experience a moderate increase, while cholesterol levels significantly decline. This reduction in circulating cholesterol is linked to a lower risk of coronary heart disease (CHD) [56].

### 3.10. Hepatoprotective Activity

Hyperlipidemia is also a key factor in the development of non-alcoholic fatty liver disease apart from atherosclerosis, and cardiovascular conditions [59]. Elevated liver enzymes in the serum, such as ALT, AST, ALP, LDH, and CK, are widely recognized markers of liver damage due to the leakage of cellular enzymes into the bloodstream [60,61]. Acidic-extractable indusiata polysaccharides (Ac-DPS) have shown significant potential in mitigating hyperlipidemia. The study demonstrated that high-fat emulsion treatment induced acute hepatotoxicity, as evidenced by increased serum enzyme activities. However, Ac-DPS supplementation effectively normalized these enzymatic activities, highlighting its protective role [9]. Furthermore, elevated total bilirubin (TBIL) levels, another indicator of liver damage, were significantly reduced following Ac-DPS administration, demonstrating its hepatoprotective effects [62]. Histopathological analysis revealed severe hepatic impairment, such as fatty vacuolation and ballooning degeneration, in the high-fat emulsion-treated group. Remarkably, Ac-DPS supplementation, especially at 400 mg/kg bw, restored liver tissue integrity, underscoring its ability to attenuate liver damage caused by hyperlipidemia [9]. Oxidative stress, driven by excessive reactive oxygen species (ROS), accelerates hyperlipidemia progression [63]. Ac-DPS treatment restored antioxidant enzyme activities (SOD, CAT, and GSH-Px) and reduced oxidative damage markers (MDA and LPO), confirming its ability to alleviate hepatic and renal oxidative stress. Furthermore, Ac-DPS improved renal function, as evidenced by normalized CREA, UREA, and ALB levels, and mitigated tubular injury in hyperlipidemic rats [13].

### 3.11. Wound Healing Activity

Matrix metalloproteinases (MMPs), a family of zinc-dependent endopeptidases, are consistently reported to be elevated in chronic wounds. These enzymes can degrade nearly all components of the extracellular matrix (ECM) and are produced by various skin cells, including fibroblasts, keratinocytes, macrophages, endothelial cells, mast cells, and eosinophils. MMP activity is regulated by tissue inhibitors of metalloproteinases (TIMPs) [64,65]. In chronic wounds, particularly in diabetic patients, elevated levels of pro-MMP-2, MMP-2, MMP-8, and MMP-9 result in an imbalance between MMPs and TIMP-2, contributing to poor wound healing [66]. Notably, MMP-2 expression is significantly higher in hypertrophic scars and keloids compared to non-scarred tissues [67]. Reducing MMP-2 expression has been shown to restore collagen types I and III, promoting faster wound healing [68]. In a study of gelatin zymography investigating the inhibitory effects of *D. indusiata* on MMP-2 activity in human skin fibroblasts, three extracts were evaluated: the peel and green mixture (PGW), the core (CW), and the whole mushroom (WW). Among these, PGW demonstrated the highest inhibition of MMP-2 activity, with a reduction of 59.63 ± 8.31%. CW followed with 41.33 ± 9.44%, while WW exhibited the lowest inhibitory effect at 16.33 ± 2.91%. These findings suggest that *D. indusiata*, particularly the PGW extract, may have potential therapeutic benefits in managing inflammatory skin conditions and reducing scar formation by modulating MMP-2 activity [10].

### 3.12. Toxicity Assessment

The diverse bioactive components present in *D. indusiata* are key contributors to its wide range of biological and physiological activities. As far as toxicity is concerned, *D. indusiata* is widely consumed in countries like China and Japan without toxicity concerns, except when excessive extracts or isolated compounds are ingested. Studies in mice show no toxic effects or behavioral changes at doses up to 1200 mg/kg for two months [1]. An acute toxicity study was conducted using eighteen male Kunming strain mice, which were divided into three groups. Two dose groups received Ac-DPS at 1000 mg/kg and 1500 mg/kg body weight, while the control group received saline. Mice had unrestricted access to food and water and were monitored for any signs of mortality or behavioral changes. The results showed no significant toxic effects, behavioral alterations, or deaths in the Ac-DPS treated groups compared to the control, indicating that Ac-DPS is virtually non-toxic under the tested conditions [9].

## 4. General Discussion and Future Perspective

Mushrooms are celebrated worldwide for their distinctive flavor and nutritional value, making them a culinary delight. Of over 2000 species, only about 25 are widely accepted as food, with several commercially cultivated. They hold dual roles as both edible and medicinal, offering organoleptic, medicinal, and nutraceutical benefits. Many edible species also exhibit therapeutic properties, blurring the distinction between edible and medicinal mushrooms [69]. Mushrooms possess significant health benefits due to their rich content of bioactive volatile organic compounds, including lectins, terpenoids, phenolics, polyphenolics, polysaccharides, and ergosterols. These compounds are widely recognized for their medicinal properties, demonstrating anti-diabetic, anti-hypercholesterolemic, antioxidant, anti-tumor, hepatoprotective, and anti-bacterial effects, which contribute to promoting overall human health. Their therapeutic potential positions them as valuable agents in the prevention and management of various diseases, underscoring the importance of mushrooms as functional foods and sources of bioactive substances in health promotion and disease prevention strategies [70]. Polysaccharides are natural polymers composed of aldoses or ketoses connected by glycosidic bonds. These complex carbohydrates play a crucial role in sustaining various life functions, serving as essential biological macromolecules within living organisms [23,29].

Among reactive oxygen species, hydroxyl radicals are highly reactive and can cause severe damage to biomolecules in living cells. Hydrogen peroxide and superoxide can indirectly generate hydroxyl radicals via Fenton or iron-catalyzed Haber–Weiss reactions, leading to oxidative injury. Antioxidants can inhibit these processes, protecting cells and food systems. Superoxide anions, precursors to other reactive oxygen species, induce oxidative damage to lipids, proteins, and DNA. DPPH, a stable free radical, is commonly used to assess antioxidant capacity, with reduced absorbance at 517 nm indicating effective radical scavenging. The prevalence of neurodegenerative diseases, marked by the gradual loss of neurons, is on the rise. Central to the pathogenesis of these conditions is oxidative stress, particularly the excessive production of reactive oxygen species (ROS). In vivo studies have revealed products of lipid, protein, and DNA oxidation associated with these diseases. While ROS plays essential roles in cell signaling and metabolic regulation, their highly reactive nature makes them detrimental to biological materials. Neurons are especially vulnerable to oxidative damage due to their high content of polyunsaturated fatty acids, limited antioxidant defenses, and elevated oxygen consumption. Therefore, the excessive ROS production in neurons is particularly harmful, involving complex mechanisms that lead to the oxidative degradation of vital biomolecules [71]. *D. indusiata* shows neuroprotective potential due to bioactive compounds like polysaccharides, dictyophorines A and B, and quinazoline compounds. These compounds enhance nerve growth factor synthesis, mitigate oxidative stress, improve mitochondrial function, and protect against neuronal apoptosis, synaptic dysfunction, and glutamatergic neurotransmission damage.

Oxidative eustress, defined by physiological levels of reactive oxygen and nitrogen species (ROS/RNS), plays a crucial role in various biochemical processes, including signal transduction pathways like NF-κB and MAPK. However, elevated ROS/RNS levels from endogenous and exogenous sources lead to oxidative stress, a common factor in numerous chronic diseases. The ROS and RNS cause oxidative damage to DNA, proteins, and lipids and increase oxidative stress biomarkers. Conditions such as cancer, cardiovascular diseases, diabetes, neurological disorders, and aging are linked to oxidative stress. Antioxidants, including enzymes and low-molecular-weight compounds, counteract oxidative damage, with vitamin E serving as a key protector against lipid peroxidation. Emerging research suggests that certain antioxidants may enhance cellular defenses and act as potential anti-cancer agents [72]. The polysaccharides from *D. indusiata* (DIPs) play a crucial role in antioxidant, gut-health-promoting, and immunomodulatory effects. Purified water-soluble polysaccharides (PPS) from *D. indusiata* demonstrate significant antioxidant activity, scavenging free radicals and protecting against oxidative stress.

Additionally, DIPs improve gut microbiota balance, reversing antibiotic-induced dysbiosis and enhancing beneficial bacteria. Their anti-inflammatory properties are also notable, as they reduce pro-inflammatory cytokines and restore intestinal barrier integrity. Moreover, DIPs activate immune responses by promoting macrophage function and regulating key pathways like NF-κB. These benefits are also seen in other mushrooms such as *Agrocybe cylindracea*, *Cordyceps sinensis*, *Trametes versicolor*, *Ganoderma lucidum*, and *Pleurotus eryngii*, supporting the beneficial outcomes of *D. indusiata*. Edible and medicinal mushrooms exhibit health-promoting properties due to their bioactive compounds, particularly polysaccharides like *β*-glucans. These compounds possess anti-obesity, anti-diabetic, anti-carcinogenic, anti-microbial, and anti-viral effects, while also offering antioxidative, anti-inflammatory, and immunomodulating benefits. Mushroom polysaccharides act as prebiotics, influencing gut microbiota and promoting beneficial bacterial growth. Their resistance to digestion allows them to impact various health conditions [73,74,75,76]. *D. indusiata* polysaccharides (DIP) have potential medicinal benefits for metabolic disorders. A study of polysaccharide fractions from *D. indusiata* tested their effects on metabolic syndrome (MetS) in mice and showed that all fractions significantly improved obesity, hypertension, dyslipidemia, and liver damage. Additionally, D6P and D8P fractions helped regulate abnormal glucose metabolism. Furthermore, other studies indicate that mushrooms such as *Agaricus bisporus* and *Pleurotus ostreatus* may have beneficial effects on various cardiometabolic risk factors. Consuming either whole or processed *A. bisporus* mushrooms has been shown to positively impact glucose levels, lipid profiles (including total cholesterol, HDL, LDL, and triglycerides), and several inflammatory markers, such as TNF-α and adiponectin. Additionally, these mushrooms enhance antioxidant capacity, as evidenced by an increase in oxygen radical absorbance capacity (ORAC) [77].

Macrophages are critical players in immune defense and inflammation, activated by external stimuli to produce nitric oxide (NO) and cytokines such as TNF-α, IL-1, IL-6, and IL-12 [78,79]. DIP can stimulate macrophage activation, enhancing the production of NO and pro-inflammatory cytokines in vitro, consistent with previous studies [5,19,80]. This activation is largely mediated by the TLR4 receptor, as shown by the binding of DIP to macrophages and the inhibition of DIP-induced cytokine production via anti-TLR4 mAb [81,82]. Additionally, TLR4-mediated macrophage activation is linked to NF-κB signaling, and DIP significantly upregulates NF-κB p65 expression, further supporting its immunomodulatory effects [83,84]. These results highlight the role of DIP in enhancing immune response through TLR4 and NF-κB pathways, offering potential for therapeutic applications in immune regulation. Lectins, found in various plants, fungi, or other species, are proteins with the ability to bind carbohydrates and have potent biological activity. Once seen as toxins, they now show promise in medicine for immunomodulation, enhancing immune responses and potentially serving as vaccines to combat microbial infections through lectin-based therapies [85,86]. As described above, immunomodulatory effect of DIP has been explored; however, no studies have yet explored the immunomodulatory effects of lectins derived from *D. indusiatus*. The immunomodulatory effects of plant lectins on various immune cells are well established in the literature, highlighting their ability to enhance phagocytic activity and cytokine production during bacterial infections [87]. One of the most studied lectins in this regard is Concanavalin A (Con A), which has demonstrated significant immunomodulatory properties. Research shows that Con A treatment in murine macrophages increases the expression of Toll-like receptors (TLRs) through JNK, p38, and NF-κB-dependent signaling pathways. TLRs are key components of the innate immune system that recognize pathogen-associated molecular patterns (PAMPs) from different bacteria and activate immune responses against them. Additionally, Con A-treated macrophages secrete pro-inflammatory cytokines and nitric oxide (NO) via TLR-mediated pathways, which help to neutralize and eliminate infectious pathogens [88]. Therefore, the induction of TLRs by plant lectins like Con A is crucial for enhancing immune responses and effectively clearing infections. Inhibiting MMP-2 activity is crucial for improving wound healing and managing skin conditions. Elevated MMP-2 levels in chronic wounds and scars disrupt collagen balance, hindering healing. By reducing MMP-2 activity, as seen with mushroom extracts, inflammation and scar formation can be minimized, promoting healthier skin recovery [10].

Though scientific data on the antidiabetic effects of *D. indusiata* is currently lacking, its richness in polysaccharides, particularly β-glucans, suggests promising potential. Experiments could yield outcomes similar to those seen in other mushrooms like *Pleurotus sajor-caju* [89,90]. The β-glucans in *D. indusiata* may help prevent hyperglycemia and insulin resistance by upregulating glucose transporters (GLUT-4) and adiponectin while downregulating pro-inflammatory factors such as NF-κB and IL-6. This could enhance insulin sensitivity, similar to the effects of metformin, and reduce plasma glucose, insulin, and lipid levels, along with improving antioxidant enzyme activity and glycogen storage in the liver. While *D. indusiata* polysaccharides (DIPs) have shown significant antioxidant, neuroprotective, and immunomodulatory effects, research gaps remain, particularly in exploring the role of other bioactive compounds like lectins. Future studies could investigate the immunomodulatory effects of lectins from *D. indusiata*, similar to concanavalin A (Con A), which enhances Toll-like receptor (TLR) expression via NF-κB, JNK, and p38 signaling pathways. Exploring how DIPs modulate mitochondrial pathways and impact cellular metabolism could further unravel their neuroprotective mechanisms. Additionally, the potential synergistic effects of DIPs with other mushroom-derived compounds in managing metabolic syndrome should be examined. Mechanistic studies on quinazoline compounds’ neuroprotection through glutamate receptor modulation and the anti-apoptotic role of DIPs in synaptic protection are important and should be further investigated. Expanding research on these mechanisms can provide deeper insights into their therapeutic applications, including neurodegenerative diseases, cancer, and inflammation. Moreover, clinical trials are needed to validate preclinical findings, ensuring safety and efficacy for human use.

## 5. Conclusions

Mushrooms, including *D. indusiata*, offer significant health benefits due to their rich bioactive compounds, such as polysaccharides, terpenoids, phenolics, and ergosterols. These compounds exhibit antioxidant, anti-inflammatory, and neuroprotective properties, making them valuable in disease prevention and therapeutic applications. Polysaccharides from mushrooms, particularly *D. indusiata*, enhance immune responses, regulate oxidative stress, and restore gut health. Furthermore, they offer neuroprotective effects by mitigating oxidative damage, improving mitochondrial function, and supporting nerve growth factor synthesis. The presence of lectins and quinazoline compounds further contributes to immune modulation and protection against neurodegenerative diseases. Overall, mushrooms serve as a source of functional foods and nutraceuticals, offering therapeutic potential against chronic diseases and promoting human health.

## Figures and Tables

**Table 1:** Literature review summarizing important experimental setup, assays, and key findings.

Parts of *D. indusiata* and Extraction Method	Experimental Model/Assay	In Vivo/In Vitro	Important Findings Following Extract/Compound Treatment	References
Fruiting body; acidic-extractable polysaccharide (Ac-DPS)	Hydroxyl radical scavenging assay; DPPH radical scavenging assay; Superoxide radical scavenging assay Concentration—0.2 to 1.4 mg/mL	In vitro	Concentration-dependent scavenging of hydroxyl, superoxide, and DPPH radicals.	[9]
	High-fat emulsion diet for mice; Ac-DPS dose 400 mg/kg or 200 mg/kg for 40 days orally daily.	In vivo	Significantly decreased hepatic lipid levels (TC, TG, NEFA); reduced serum biomarkers for liver injury (ALT, AST, ALP, LDH, and CK); reduced TIBIL level; reduced serum lipid levels of TC, TG, LDL-C, and HDL-C; decreased oxidative stress-related enzymes SOD, GSH-Px, and CAT and non-enzymatic activity T-AOC in liver and kidney; lipid peroxidation level including MDA and LPO reduced; Ac-DPS mitigated the high-fat emulsion-induced kidney damage, reduced the UREA and CREA, and increased the ALB contents in serum. Reduced the INS and LEP contents but increased the ADPN level; restored the histopathological changes in the liver and kidney.	
Immature (egg) bamboo mushroom; aqueous extracts	DPPH and ABTS assay; FRAP assay; anti-inflammatory activity on LPS-induced RAW 264.7 macrophages; wound healing activity	In vitro	Scavenges reactive oxygen species; acts as good metal chelator; inhibits NO, IL-1, IL-6, and TNF-α in LPS-induced RAW 264.7 macrophages in a dose- and time-dependent manner; inhibits MMP-2 expression in hTRT fibroblasts cells.	[10]
Fruiting body; aqueous extracts	BALB/C mice given high-fat diet for 8 weeks, induced obesity. From the 4th week onwards, DIP was given at 200 mg/kg and 400 mg/kg body weight.	In vivo	DIP reduced the HFD-induced body weight, liver weight, epididymal, and subcutaneous fat; reduced expression of adipogenic genes by regulation of transcription factors such as expression level of PPAR-g, C/EBPa, and SREBP-1c; ameliorated liver health by reducing ALT, AST, TG, and FFA; reduction in glucose, insulin, and serum LPS level; improved intestinal tight junction proteins (TJP) expression including claudin-1, occludin, and zonula occluden (ZO-1); decreased pro-inflammatory cytokines TNF-α, IL-6, and IL-1β and enhanced anti-inflammatory cytokines IL-4 and IL-10 in serum; DIP treatment improved gut microbiota, level of Firmicutes decreased and Bacteroidetes increased.	[11]
Fruiting body; aqueous extracts	Hydroxyl radical scavenging assay; DPPH radical scavenging assay; superoxide radical scavenging assay; ferrous ion chelating assay, 2 mg/mL; antimicrobial activity, 200 mg/mL	In vitro	Extract displays antioxidant activity, scavenges DPPH free radicals and superoxide ions, and inhibits bacterial growth (maximum inhibition for Alcaligenes faecalis) and fungal growth (maximum inhibition for Candida albican).	[12]
Fruiting body; Aqueous extracts	Hydroxyl radical scavenging assay; DPPH free radical scavenging assay; concentration 0.2–1.0 mg/mL.	In vitro	Scavenges hydroxyl radicals and DPPH free radicals.	[13]
	High-fat emulsion-induced obese mice, diet given daily for 45 days. Aqueous extracts are given daily oral gavage of 400 mg/kg bw and 200 mg/kg bw.	In vivo	WPS treatment decreases body weight in obese mice; reduces level of TC, TG, LDL-C, and AI in serum, increases serum HDL-C level to greater than model group; reduces liver injury markers ALT, AST, ALP, LDH, and CK in serum; increases levels of hepatic antioxidant enzymes SOD, GSH-Px, and CAT and T-AOC; contents of lipid peroxide MDA, LPO, and MPO reduced as compared to obese mice; reduces hepatic lipid level TC, TG, and NEFA in treated group; reduces HI; treatment increases the renal antioxidant enzymes SOD, GSH-Px, CAT, and T-AOC; relieves renal oxidative stress by lowering MDA, LPO, and MPO contents in obese mice; reduces RI levels; kidney dysfunction parameter in serum analyzed; WPS reduces UREA and CREA level and increases ALB compared to obese mice; reduces INS and LEP and increases ADPN in treated mice; restores liver and kidney tissue damage due to obesity by WPS treatment.	
Fruiting body; aqueous extracts (PD3) and regenerated aqueous extract (RPD3)	CCK-8 assay for cell viability of mouse sarcoma S180 cells. Concentration—0.2, 0.5, and 1 mg/mL in 0.9% aqueous NaCl.	In vitro	The PD3 and RPD3 did not show direct cytotoxicity against S-180 cells. Cytotoxicity was less than 5% at a higher concentration of 1 mg/mL.	[14]
	Tumor induced in mice by inoculating ascitogenous sarcoma S180 cells; dosages 50 mg/kg, 100 mg/kg, 200 mg/kg bw. Extract injected intraperitoneally once daily for 10 d, starting 24 h after tumor inoculation.	In vivo	RPD3 shows higher tumor inhibition in comparison to PD3 in dose-dependent manner. PD3 and RPD3 both show protective effects on the thymus and spleen. Cytokines IL-2, IL-6, and TNF-α in serum were upregulated, which causes immune stimulation to promote anti-tumor activity.	
Fruiting body; 85% ethanolic extract	Isolated compounds Dictyophorine A and Dictyophorine B were tested on quiescent rat astroglial cells for NGF synthesis.	In vitro	Dictyophorine A induces NGF synthesis four times more in quiescent rat astroglial cells than untreated cells. Dictyophorine B also significantly induces synthesis of NGF, but less than Dictyophorine A.	[15]
Fresh mushroom; methanolic extract	Isolated compounds dictyoquinazols A, B, and C tested for neuroprotective activity on mouse cortical neurons. Concentration—10–30 μM.	In vitro	Dictyoquinazols protect mouse cortical neurons from glutamate- and NMDA-induced excitotoxicities in a dose-dependent manner but could not protect neurons from AMPA and kainite.	[16]
Fruiting body; dichloromethane extract	Isolated sesquiterpene antibiotic albaflavenone	In vitro	Albaflavenone is an anti-bacterial compound isolated from *D. indusiata*; the presence of albaflavenone contributes to its anti-bacterial activity mentioned elsewhere.	[17]
Fruiting body; Aqueous extracts	Water-soluble polysaccharide (DI), checked for immunomodulatory activities on RAW 264.7 macrophage.	In vitro	DI is a β-(1→3)-glucan with side branches of β-(1→6)-glucosyl units, and it has a triple-helical structure; DI is not cytotoxic to cells. It induces NO, TNF-α, IL-1, IL-6, and IL-12 production which promotes macrophage multiplication.	[18]
Fruiting body; aqueous extracts	MTT assay—50–200 µg/mL (to determine the effect of DIP on cell proliferation of RAW 264.7 cells); nitric oxide (NO) production assay; ELISA assay; RT-PCR; cytokine production—50–200 µg/mL.	In vitro	DIP enhances the proliferation of RAW 264.7 cells; DIP induces the up-regulation of NO, IL-1β, IL-6, and TNF-α in RAW 264.7 in a concentration-dependent manner. RT-PCR indicates that DIP up-regulated the mRNA expression of iNOS, IL-1β, IL-6, and TNF-α; anti-TLR4 inhibits the production of NO and cytokines, indicating TLR4 was involved in the DIP-induced macrophage activation; DIP upregulates NF-κB, indicating that macrophage activation involved NF-κB signaling.	[19]
Fruiting body; aqueous extracts (isolated DIP was a homogeneous β-(1→3)-D-glucan with side branches of β-(1→6)-glucosyl units).	Acute colitis was induced in C57BL/6 mice by feeding 2.5% dextran sulfate sodium (DSS) in drinking water for 7 days. Dose—25 mg/kg/d, 50 mg/kg/d and 100 mg/kg/d.	In vivo	DIP improves weight loss due to DSS; relieves splenomegaly; reduces histopathologic damage such as intestinal crypts and goblet cells, tissue architecture, and inflammatory infiltration; relives intestinal oxidative stress; increases the level of GSH; decreases the level of MDA; upregulates HO-1 protein expression; gene expression of IL-10 upregulated; gene expression suppressed for TNF-α, IL-6, and IL-1β; reduces MPO activity. DIP abolished the colitis-induced elevated expression of NLRP3, phosphorylated-STAT3, and p-IκBα; upregulated Bcl2 and downregulated Bax; inhibited DSS-induced lower TJP-1 protein expression in colonic tissues.	[20]

Table abbreviations: MMP—Matrix Metalloproteinase; DPPH—2,2-Diphenyl-1-picrylhydrazyl; TC—Total Cholesterol; TG—Triglycerides; NEFA—Non-Esterified Fatty Acids; ALT—Alanine Aminotransferase; AST—Aspartate Aminotransferase; ALP—Alkaline Phosphatase; LDH—Lactate Dehydrogenase; CK—Creatine Kinase; TIBIL—Total Bilirubin; LDL-C—Low-Density Lipoprotein Cholesterol; HDL-C—High-Density Lipoprotein Cholesterol; SOD—Superoxide Dismutase; GSH-Px—Glutathione Peroxidase; CAT—Catalase; T-AOC—Total Antioxidant Capacity; MDA—Malondialdehyde; LPO—Lipid Peroxidation; UREA—Urea; CREA—Creatinine; ALB—Albumin; INS—Insulin; LEP—Leptin; ADPN—Adiponectin; NO—Nitric Oxide; IL—Interleukin; TNF-α—Tumor Necrosis Factor-alpha; PPAR-γ—Peroxisome Proliferator-Activated Receptor Gamma; C/EBPα—CCAAT/Enhancer-Binding Protein Alpha; SREBP-1c—Sterol Regulatory Element-Binding Protein 1c; TJP—Tight Junction Protein; ZO-1—Zonula Occludens 1; FRAP—Ferric Reducing Antioxidant Power; LPS—Lipopolysaccharide; DIP—*Dictyophora indusiata* Polysaccharide; PD3—Polysaccharide Dictyophora Extract 3; RPD3—Regenerated Polysaccharide Dictyophora Extract 3; NGF—Nerve Growth Factor; AMPA—α-Amino-3-hydroxy-5-methyl-4-isoxazolepropionic acid; NMDA—N-Methyl-D-Aspartate; CRE—Creatinine; DSS—Dextran Sulfate Sodium; RT-PCR—Reverse Transcription Polymerase Chain Reaction; NF-κB—Nuclear Factor Kappa B; iNOS—Inducible Nitric Oxide Synthase; TLR4—Toll-Like Receptor 4; Bcl2—B-cell Lymphoma 2; Bax—Bcl2-Associated X Protein; NLRP3—NOD-Like Receptor Pyrin Domain Containing 3; STAT3—Signal Transducer and Activator of Transcription 3; p-IκBα—Phosphorylated Inhibitor of Nuclear Factor Kappa B Alpha; HO-1—Heme Oxygenase-1.

## Data Availability

No new data were created or analyzed in this study.
